# Learning strategies, study behaviors, and academic performance among medical students in Jordan: A cross-sectional study

**DOI:** 10.1371/journal.pone.0348623

**Published:** 2026-06-10

**Authors:** Wafaa A. Mahmoud, Ahmad A. Alrawashdeh, Khaled A. Mahmoud, Nasr N. Alrabadi, Ibrahim M. Hoja, Abdelrahman M. Qasaymeh, Abdulla M. AlKofahi, Mohammad M. Alsmadi, Basel A. Alnajjar

**Affiliations:** 1 Department of Anatomy, Faculty of Medicine, Jordan University of Science and Technology, Irbid, Jordan; 2 Department of Allied Medical Science, Faculty of Applied Medical Science, Jordan University of Science and technology, Irbid, Jordan; 3 Heart Center, Department of Cardiology, Angiology, Pneumology and Medical Intensive Care, University Hospital Bonn, Venusberg-Campus 1, Bonn, Germany; 4 Department of Pharmacology, Faculty of Medicine, Jordan University of Science and Technology, Irbid, Jordan; 5 Faculty of Medicine, Jordan University of Science and Technology, Irbid, Jordan; Mutah University, JORDAN

## Abstract

Academic performance in medical education is influenced by multiple factors, including learning strategies and study behaviors. However, the extent to which these factors are associated with academic performance remains unclear. This study aimed to describe the frequency of learning strategies and examine their associations with academic performance among medical students, with emphasis on evidence-based strategies. A cross-sectional anonymous online survey was conducted among medical students at Jordan University of Science and Technology. Learning strategies were assessed using ten items rated as rarely, sometimes, or often. Composite indices were constructed for overall, evidence-based, and common/passive strategies. Academic performance was categorized as excellent, very good, or good or below. Group comparisons were performed using chi-square tests and ANOVA, and multivariable logistic regression identified factors associated with excellent performance. Among 556 respondents, 52.2% reported excellent performance. Evidence-based strategies showed a graded increase across performance groups and were independently associated with higher odds of excellent performance (aOR 1.47, 95% CI 1.16–1.86), whereas common/passive strategies were not. These findings suggest that evidence-based learning strategies are associated with better academic performance, supporting their potential role in medical education. However, the findings are exploratory rather than causal.

## Introduction

Medical education requires a large volume of complex information to be learned within a limited timeframe [1]. To meet these requirements, students must be self-regulated, strategic, and motivated to perform higher-order cognitive tasks, enhance successful learning, and generate retrievable memory [2]. Prior academic achievement, personal characteristics, and cognitive strategy use are among the factors that shape academic performance in medical education [3,4]. It has been suggested that students’ preferred learning strategies account for about half of the difference in examination performance among pre-clinical medical students [5].

Self-regulated learning provides an important theoretical background for understanding how students actively manage their learning processes [2,6]. Self-regulated learning integrates cognitive, metacognitive, motivational, and behavioral components through which learners plan learning activities, manage time effectively, regulate motivation for learning, monitor their learning progress, and regulate the use of effective learning strategies [2,6]. Several self-regulated learning models have been proposed to explain how students regulate their learning processes [6]. Prominent models developed by Zimmerman, Pintrich, Winne and Hadwin conceptualize learning as a cyclical process in which students set goals, select learning strategies, monitor their progress, and reflect on outcomes to improve future performance [6]. Within this framework, learning strategies represent key mechanisms through which students regulate their cognitive processes and are hypothesized to influence academic performance [7].

Among the various self-regulated learning models, the framework proposed by Pintrich offers a comprehensive, structured model that is particularly appropriate for educational research [6]. Pintrich conceptualizes self-regulated learning as a cyclical process consisting of four phases: forethought, monitoring, control, and reflection. These phases operate across multiple domains, including cognition, motivation, behavior, and learning context, providing an integrative model of how students regulate their learning processes [6]. Pintrich further proposed that self-regulatory processes mediate the relationship between learners and their learning environment, thus influencing academic achievement [8]. Within this framework, learning strategies are a key element of cognitive regulation, reflecting how students process, organize, and apply information during learning. In addition, the model includes behavioral regulation (e.g., time management) and motivational regulation, both of which are essential in demanding educational environments such as medical education [6]. Within this view, learning strategies are hypothesized to influence academic performance by affecting cognitive processing, metacognitive monitoring, and the regulation of study behavior. Pintrich’s model, therefore, provides a suitable theoretical basis for examining these associations.

Within this context, different approaches to learning have been described to explain how students engage with academic material. Three principal learning strategies that are widely applicable in education have been extensively investigated and applied among medical students and practicing physicians [9]. Surface learning involves relying on memorization of key facts rather than developing a deep understanding of the subject [10,11]. Another is deep learning, which is driven by students’ interest in gaining a deeper understanding of the subject [11,12]. Lastly, using a strategic approach to learning, in which students are motivated by academic success, prompts students to tailor their study methods to assessment requirements [10].

While several learning strategies have been recommended and assessed in the literature, we focus on those identified as particularly effective. These include evidence-based strategies which have been evaluated in the psychology literature, such as spaced practice (studying over time), interleaving (switching topics within a session), retrieval practice (recalling information from memory), elaboration (explaining how and why things work), dual coding (using verbal and visual forms), and the use of concrete examples (applying concepts to real-life situations), as well as commonly used strategies such as summarization (writing summaries of texts), highlighting/underlining (marking key reading material), keyword mnemonics (linking keywords and mental images), and rereading (reviewing text after an initial reading) [1,13,14]. Although students commonly employ many learning strategies, some are ineffective [10]. The wide variety of existing strategies makes it challenging for students to identify effective, practical approaches [13].

In addition to learning strategies, efficient time management in medical education has been linked to improved academic achievement [15–18]. In demanding medical education, students must manage their time efficiently to complete required tasks and acquire the necessary knowledge within a limited timeframe [19]. Establishing clear educational goals and developing structured study plans are essential to achieving academic success, which includes adhering to organized course schedules, effective exam preparation, and leaving enough time for other essential daily activities [19].

Despite evidence from cognitive psychology, the use of learning strategies among medical students remains variable [1]. Several factors affect strategy selection, including personal characteristics, learning environments, and conceptions of learning [20–22]. However, the extent to which specific evidence-based learning strategies are associated with academic performance, independent of study behaviors and contextual factors, remains insufficiently characterized in medical education settings. Therefore, this study aimed to assess the frequency of key learning strategies and study behaviors among medical students at Jordan University of Science and Technology (JUST), with a focus on the association of evidence‑based and common strategies with academic performance. Accordingly, the study addressed the following research questions: which learning strategies are most frequently used by medical students, and whether the frequency of evidence-based and commonly used strategies differs across academic performance groups.

## Materials and methods

### Study design and setting

A cross-sectional, questionnaire-based study was conducted among medical students at JUST in Irbid, Jordan, using an anonymous, self-administered questionnaire through Google Forms. The study was approved by the local Institutional Review Board at JUST (IRB) (Ref. No. June 2025/183–46). Data were collected between July 1, 2025, and August 25, 2025. All participants’ information was anonymized prior to analysis, and the authors had no access to any data that could identify individual participants during or after data collection.

### Participants and recruitment procedures

Eligible participants were students enrolled in the medical program at JUST across all academic years from first to sixth year, and participation was voluntary. The survey was anonymous, and no identifying information was collected. The survey link was distributed electronically through institutional email lists and student communication channels, including Facebook and WhatsApp student groups. The invitation targeted medical students across all academic years. Participation was voluntary, and no identifying information was collected. A consent statement was displayed on the first page of the questionnaire; students proceeded only after confirming their consent. The average completion time was approximately 10–15 minutes.

The minimum required sample size was estimated using the single-population proportion formula, assuming a 95% confidence level, a 5% margin of error, and an expected proportion of 50%. The 50% assumption was used because it provides the most conservative estimate when the expected prevalence of learning-strategy use is unknown. Based on these assumptions, the minimum required sample size was 385 students. The final sample included 556 respondents, exceeding the minimum required sample size.

### Survey instrument and study variables

The questionnaire comprised 20 structured multiple-choice items covering demographic characteristics, preferred study methods and learning strategies, time management, and exam preparation behaviors. The questionnaire was developed based on commonly described learning strategies in cognitive psychology and medical education literature. Academic experts evaluated each questionnaire item for clarity, relevance, content coverage, and wording, and their feedback was used to improve the survey. A convenience sample of 10 medical students then completed the questionnaire and took part in brief cognitive interviewing sessions to identify unclear or repetitive items. As each question required a response before advancing to the next item, no missing data were present in the datasets.

The questionnaire was designed to capture a broad range of learning strategies and behaviors and was not intended as a fully validated psychometric scale. Accordingly, the findings should be interpreted as exploratory and hypothesis-generating. An English-language version is provided as File S1 File: Study questionnaire.

Academic performance was self-reported and referenced the most recent academic year for students in the second to sixth years and the most recent semester for first-year students, using the official grading scale of Jordan University of Science and Technology, in which excellent corresponds to grades A or A + , very good corresponds to grade B, good to grade C, fair to grade D, and poor to grade F.

### Learning strategy measures

Learning strategy items captured a set of evidence-based strategies [1,14], including spaced practice, interleaving, retrieval practice, elaboration, concrete examples, and dual coding, as well as commonly used strategies [13], including summarization, highlighting or underlining, keyword mnemonic technique, and rereading. Each learning-strategy item was scored numerically as 1 for rarely, 2 for sometimes, and 3 for often, and indices were calculated as mean scores to retain the original 1–3 metric for interpretability across indices.

The overall strategies score was the mean of all 10 items. The evidence-based strategies score was the mean of six items: spaced practice, interleaving, retrieval practice, elaboration, concrete examples, and dual coding. The common/passive strategies score was the mean of four items: summarization, highlighting, keyword mnemonic, and rereading. Higher scores reflected more frequent use.

### Covariates and study behaviors measures

Covariates were selected based on theoretical relevance to self-regulated learning frameworks, including factors related to academic context (year of study, academic phase), demographic characteristics, and study behaviors that may influence the use of learning strategies and academic performance. Academic context was represented using the year of study and a derived academic phase variable, with the preclinical phase defined as years 1–3 and the clinical phase as years 4–6. Study behaviors included daily study hours, time management techniques, timing of preparation for major examinations, prioritized exam resources, typical study patterns, and primary study focus.

### Statistical analysis

All analyses were conducted using Stata (StataCorp LLC, College Station, TX). Categorical variables were summarized as frequencies and percentages (%), and continuous variables and strategy indices were summarized using means and standard deviations (SDs). The distribution of composite strategy indices was examined using histograms, Q-Q plots, and descriptive statistics. Because the distributions showed only modest deviation from normality and the sample size was large, ANOVA was used to compare mean composite scores across academic performance categories. Kruskal-Wallis tests were additionally conducted as sensitivity analyses to examine the robustness of the findings. We grouped academic performance into excellent, very good, good or below for descriptive and bivariate comparisons. For descriptive analyses, differences across the three academic performance categories were evaluated using Pearson’s chi-square test, and Fisher’s exact test was used when sparse expected counts required exact inference.

For multivariable modelling, we dichotomized academic performance as excellent versus very good or below. We first fitted univariable logistic regression models to estimate crude odds ratios (ORs) and 95% confidence intervals (CIs). Next, we fitted a multivariable logistic regression model to estimate adjusted odds ratios (aORs) and 95% CIs for factors linked to excellent performance. In this model, we controlled for demographics and study behaviors. We standardized the strategy indices (z-scores) and entered them in 1-SD increments for comparability. We summarized model fit using McFadden’s pseudo-R². All tests were two-sided, with p < 0.05 considered significant.

### Ethics approval and consent to participate

The study was approved by the local Institutional Review Board (IRB) at Jordan University of Science and Technology (JUST), Irbid, Jordan (Ref. No. June 2025/183–46) and was conducted in accordance with the ethical principles outlined in the Declaration of Helsinki. Participation was voluntary and preceded by an electronic informed consent form. The study’s confidentiality was maintained through an anonymized survey design and by avoiding direct identifiers.

## Results

### Participant characteristics and the distribution of academic performance

A total of 556 students participated, corresponding to a response rate of approximately 14%; of these, 290 (52.2%) reported excellent academic performance, 146 (26.3%) reported very good performance, and 120 (21.6%) reported good or below. Characteristics and study behaviors by performance category are shown in Table 1.

**Table 1:** Participant characteristics and study behaviors by academic performance category.

	Overall N = 556	Good or below n = 120	Very good n = 146	Excellent n = 290	p-value*
**Gender**					0.830
Male	199 (35.8)	41 (34.2)	55 (37.7)	103 (35.5)	
Female	357 (64.2)	79 (65.8)	91 (62.3)	187 (64.5)	
**Academic phase**					0.017
Pre-clinical (1^st^, 2^nd^, and 3^rd^ years)	268 (48.2)	65 (54.2)	56 (38.4)	147 (50.7)	
Clinical (4^th^, 5^th^, and 6^th^ years)	288 (51.8)	55 (45.8)	90 (61.6)	143 (49.3)	
					
**Study hours/day**					0.076
<2 hours	61 (11.0)	18 (15.0)	22 (15.1)	21 (7.2)	
2–4 hours	153 (27.5)	37 (30.8)	39 (26.7)	77 (26.6)	
4–6 hours	223 (40.1)	43 (35.8)	52 (35.6)	128 (44.1)	
>6 hours	119 (21.4)	22 (18.3)	33 (22.6)	64 (22.1)	
**Time management tool**					0.002
No specific tool	170 (30.6)	42 (35)	40 (27.4)	88 (30.3)	
To-do lists/planner	211 (38)	26 (21.7)	60 (41.1)	125 (43.1)	
Pomodoro technique	94 (16.9)	26 (21.7)	28 (19.2)	40 (13.8)	
Prioritization	81 (14.6)	26 (21.7)	18 (12.3)	37 (12.8)	
**Preparing for major exams**					0.001
<1 week	65 (11.7)	22 (18.3)	19 (13.0)	24 (8.3)	
1–2 weeks	180 (32.4)	42 (35.0)	55 (37.7)	83 (28.6)	
2–4 weeks	177 (31.8)	36 (30.0)	47 (32.2)	94 (32.4)	
>1 month	134 (24.1)	20 (16.7)	25 (17.1)	89 (30.7)	
**Exam resources prioritized**					0.016
Lecture slides and notes	399 (71.8)	75 (62.5)	103 (70.6)	221 (76.2)	
Textbooks	26 (4.7)	4 (3.3)	6 (4.1)	16 (5.5)	
Online videos or tutorials	70 (12.6)	26 (21.7)	18 (12.3)	26 (9.0)	
Question banks	61 (11.0)	15 (12.5)	19 (13.0)	27 (9.3)	
**Study pattern**					<0.001
Irregularly	49 (8.8)	18 (15.0)	19 (13.0)	12 (4.1)	
Regular during semester	69 (12.4)	11 (9.2)	16 (11.0)	42 (14.5)	
Intensive before exams	164 (29.5)	49 (40.8)	45 (30.8)	70 (24.1)	
Both Regular + intensive	274 (49.3)	42 (35)	66 (45.2)	166 (57.2)	
**Study primary focus**					<0.001
Understanding concepts deeply	139 (25.0)	33 (27.5)	39 (26.7)	67 (23.1)	
Memorizing key facts	25 (4.5)	11 (9.2)	8 (5.5)	6 (2.1)	
Preparing for exams specifically	91 (16.4)	31 (25.8)	27 (18.5)	33 (11.4)	
Balancing both understanding and memorization	301 (54.1)	45 (37.5)	72 (49.3)	184 (63.5)	

* Chi square test

Gender distribution did not differ across groups (p = 0.830), whereas the clinical phase was more frequent among very good students than among excellent, good, or below (61.6% vs 49.3% vs 45.8%; p = 0.017, respectively).

### Study behaviors and exam‑related behaviors by academic performance

Distributions of study behaviors (study hours, time management, study patterns, study focus, and exam preparation behaviors) across academic performance categories are summarized in Table 1. With respect to study behaviors, daily study hours did not differ significantly across performance groups (p = 0.076). In contrast, study behaviors differed with respect to time management tool use (p = 0.002), timing of exam preparation (p = 0.001), prioritized exam resources (p = 0.016), study pattern (p < 0.001), and primary study focus (p < 0.001). Combining regular semester-long study with intensive pre-exam study was most frequent among excellent performers compared with very good and good or below (57.2% vs 45.2% vs 35.0%, respectively), and balancing understanding with memorization was also most common in the excellent group (63.5% vs 49.3% and 37.5%, respectively).

### Frequency of individual learning strategies by academic performance

The frequency of individual learning strategies (rarely/sometimes/often) by academic performance category is shown in Table 2. Several evidence-based learning strategies showed significant variation across academic performance categories. Students with excellent performance more frequently reported using spaced practice more often than students with very good or good or below performance (49.0% vs 29.5% vs 30.8%; p < 0.001, respectively). A similar gradient was observed for elaboration, with 71.0% of excellent performers reporting often using elaboration compared with 51.4% and 54.2% of the very good and good or below groups, respectively (p < 0.001). Dual coding also differed across groups (p = 0.033), with 52.1% of excellent performers reporting frequent use. In contrast, no statistically significant differences were observed for interleaving (p = 0.460), retrieval practice (p = 0.206), or the use of concrete examples (p = 0.213).

**Table 2:** Frequency of individual learning strategies by academic performance category.

	Overall N = 556	Good or below n = 120	Very good n = 146	Excellent n = 290	p-value*
**Evidence-based strategies**					
**Spaced practice**					<0.001
Rarely	131 (23.6)	32 (26.7)	37 (25.3)	62 (21.4)	
Sometimes	203 (36.5)	51 (42.5)	66 (45.2)	86 (29.7)	
Often	222 (39.9)	37 (30.8)	43 (29.5)	142 (49.0)	
**Interleaving**					0.460
Rarely	140 (25.2)	30 (25)	40 (27.4)	70 (24.1)	
Sometimes	217 (39)	54 (45)	55 (37.7)	108 (37.2)	
Often	199 (35.8)	36 (30)	51 (34.9)	112 (38.6)	
**Retrieval practice**					0.206
Rarely	85 (15.3)	25 (20.8)	23 (15.8)	37 (12.8)	
Sometimes	210 (37.8)	45 (37.5)	59 (40.4)	106 (36.6)	
Often	261 (46.9)	50 (41.7)	64 (43.8)	147 (50.7)	
**Elaboration**					<0.001
Rarely	29 (5.2)	12 (10)	6 (4.1)	11 (3.8)	
Sometimes	181 (32.6)	43 (35.8)	65 (44.5)	73 (25.2)	
Often	346 (62.2)	65 (54.2)	75 (51.4)	206 (71)	
**Concrete examples**					0.213
Rarely	62 (11.2)	19 (15.8)	17 (11.6)	26 (9)	
Sometimes	192 (34.5)	45 (37.5)	47 (32.2)	100 (34.5)	
Often	302 (54.3)	56 (46.7)	82 (56.2)	164 (56.6)	
**Dual coding**					0.033
Rarely	97 (17.5)	28 (23.3)	26 (17.8)	43 (14.8)	
Sometimes	203 (36.5)	47 (39.2)	60 (41.1)	96 (33.1)	
Often	256 (46)	45 (37.5)	60 (41.1)	151 (52.1)	
**Common/passive strategies**					
**Summarization**					0.844
Rarely	201 (36.2)	40 (33.3)	55 (37.7)	106 (36.6)	
Sometimes	200 (36)	44 (36.7)	55 (37.7)	101 (34.8)	
Often	155 (27.9)	36 (30)	36 (24.7)	83 (28.6)	
**Highlight underline**					0.644
Rarely	65 (11.7)	15 (12.5)	14 (9.6)	36 (12.4)	
Sometimes	132 (23.7)	33 (27.5)	36 (24.7)	63 (21.7)	
Often	359 (64.6)	72 (60)	96 (65.8)	191 (65.9)	
**Keyword mnemonic**					0.820
Rarely	69 (12.4)	15 (12.5)	16 (11)	38 (13.1)	
Sometimes	197 (35.4)	39 (32.5)	57 (39)	101 (34.8)	
Often	290 (52.2)	66 (55)	73 (50)	151 (52.1)	
**Rereading**					0.001
Rarely	55 (9.9)	22 (18.3)	13 (8.9)	20 (6.9)	
Sometimes	187 (33.6)	45 (37.5)	55 (37.7)	87 (30)	
Often	314 (56.5)	53 (44.2)	78 (53.4)	183 (63.1)	

* Chi square test

Among common/passive strategies, rereading differed across performance categories (p = 0.001); frequent rereading was reported by 63.1% of excellent performers compared with 53.4% of the very good group and 44.2% of the good or below group. By comparison, summarization (p = 0.844), highlighting/underlining (p = 0.644), and keyword mnemonic use (p = 0.820) were common but were not significantly associated with academic performance.

### Composite learning strategy indices

The composite indices for learning strategies (overall, evidence‑based, and common/passive) are reported in Table 3. Composite learning strategy indices showed an association between more frequent use of learning strategies and excellent academic performance. The overall mean strategy score differed across academic performance categories, increasing from 2.23 (SD ± 0.41) in the good or below group to 2.28 (SD ± 0.35) in the very good group and 2.37 (SD ± 0.32) in the excellent group (p < 0.001). The evidence-based strategies index showed a similar pattern (2.20, SD ± 0.44 vs 2.26, SD ± 0.38 vs 2.39, SD ± 0.34; p < 0.001, respectively). In contrast, the common/passive strategies index did not differ statistically across groups (p = 0.343).

**Table 3:** Composite learning strategy indices by academic performance category.

	Total Mean (SD)	Good or below Mean (SD)	Very good Mean (SD)	Excellent Mean (SD)	P-value*
All strategies (mean 1–3)	2.32 (0.35)	2.23 (0.41)	2.28 (0.35)	2.37 (0.32)	<0.001
Evidence-based strategies score (mean 1–3)	2.31 (0.38)	2.20 (0.44)	2.26 (0.38)	2.39 (0.34)	<0.001
Common/passive strategies score (mean 1–3)	2.33 (0.46)	2.28 (0.52)	2.32 (0.44)	2.35 (0.43)	0.343

* ANOVA

### Multivariable model for predictors of excellent academic performance

Factors independently associated with excellent performance versus very good or below are presented in the multivariable logistic regression model in Table 4. In logistic regression models predicting excellent performance versus very good or below, greater use of evidence-based strategies was associated with a higher odds ratio for excellent performance in both unadjusted and adjusted analyses. Specifically, each 1-SD increase in the evidence-based strategies score was associated with higher odds of excellent performance after adjustment for demographic characteristics and study behaviors (aOR 1.47, 95% CI 1.16–1.86; p = 0.001). The common/passive strategies score was not independently associated with excellent performance after adjustment (aOR 0.91, 95% CI 0.72–1.14; p = 0.417).

**Table 4:** Univariable and multivariable logistic regression of factors associated with academic performance.

	Unadjusted		Adjusted	
	**OR (95% CI)**	**P-value**	**aOR (95% CI)**	**P-value**
Evidence-based strategies score (per 1 SD)	1.53 (1.28, 1.82)	<0.001	1.47 (1.16, 1.86)	0.001
Common/passive strategies score (per 1 SD)	1.12 (0.95, 1.32)	0.187	0.91 (0.72, 1.14)	0.417
**Gender**				
Male	Ref		Ref	
Female	1.03 (0.72, 1.45)	0.888	0.9 (0.59, 1.38)	0.633
**Academic phase**				
Preclinical phase	Ref		Ref	
Clinical phase	0.81 (0.58, 1.13)	0.22	0.76 (0.5, 1.14)	0.185
**Study hours/day**				
<2 hours	Ref		Ref	
2–4 hours	1.93 (1.04, 3.57)	0.036	1.45 (0.7, 3.02)	0.315
4–6 hours	2.57 (1.42, 4.63)	0.002	1.71 (0.81, 3.59)	0.156
>6 hours	2.22 (1.17, 4.2)	0.015	1.47 (0.65, 3.3)	0.355
**Time management**				
To-do lists/planner	Ref		Ref	
No specific strategy	0.74 (0.49, 1.11)	0.144	1.2 (0.72, 1.98)	0.483
Pomodoro technique	0.51 (0.31, 0.83)	0.007	0.65 (0.36, 1.15)	0.14
Prioritization	0.58 (0.35, 0.97)	0.038	0.7 (0.38, 1.29)	0.255
**Preparing for major exams**				
<1 week	Ref		Ref	
1–2 weeks	1.46 (0.82, 2.62)	0.202	1.35 (0.68, 2.69)	0.391
2–4 weeks	1.93 (1.08, 3.47)	0.027	1.53 (0.73, 3.19)	0.256
>1 month	3.38 (1.82, 6.27)	<0.001	2.14 (0.96, 4.76)	0.061
**Exam resources prioritized**				
Lecture slides and notes	Ref		Ref	
Textbooks	1.29 (0.57, 2.91)	0.542	1.2 (0.45, 3.22)	0.721
Online videos or tutorials	0.48 (0.28, 0.8)	0.005	0.56 (0.3, 1.05)	0.07
Question banks	0.64 (0.37, 1.1)	0.106	0.76 (0.4, 1.44)	0.404
**Typical study Pattern**				
Irregularly	Ref		Ref	
Regular during semester	4.8 (2.13, 10.79)	<0.001	3.61 (1.39, 9.33)	0.008
Intensive before exams	2.3 (1.12, 4.72)	0.024	3.2 (1.41, 7.26)	0.005
Both Regular + intensive	4.74 (2.37, 9.49)	<0.001	4.04 (1.8, 9.04)	0.001
**Study primary focus**				
Understanding concepts deeply	Ref		Ref	
Memorizing key facts	0.34 (0.13, 0.9)	0.03	0.5 (0.16, 1.52)	0.221
Preparing for exams specifically	0.61 (0.36, 1.05)	0.075	0.8 (0.43, 1.5)	0.493
Balancing both understanding and memorization	1.69 (1.13, 2.54)	0.011	1.62 (1.01, 2.59)	0.046

After adjustment for covariates, study hours/day, earlier initiation of exam preparation, and time-management techniques were not significantly associated with excellent performance. However, study patterns remained significantly associated with excellent performance; compared with studying irregularly, studying regularly during the semester (aOR 3.61, 95% CI 1.39–9.33; p = 0.008), studying intensively before exams (aOR 3.20, 95% CI 1.41–7.26; p = 0.005), or combining regular and intensive study (aOR 4.04, 95% CI 1.80–9.04; p = 0.001) were each independently associated with higher odds of excellent performance. In addition, a primary study focus that balanced understanding and memorization was independently associated with excellent performance (aOR 1.62, 95% CI 1.01–2.59; p = 0.046) compared with a primary focus on deep conceptual understanding. Model fit was acceptable (McFadden pseudo-R² = 0.187), indicating that a considerable proportion of ‌‌variability in academic performance remains unexplained.

## Discussion

This study examined the association between learning strategies, study behaviors, and academic performance among medical students, with particular focus on evidence-based learning strategies. Students who reported more frequent use of evidence-based strategies were more likely to report excellent academic performance, even after adjustment for demographic characteristics and study behaviors. This suggests that engagement with learning content and active processing of information may be associated with academic performance. In contrast, commonly used or more passive learning strategies were not independently associated with higher academic outcomes. This study extends prior research by simultaneously examining evidence-based and commonly used strategies within a single analytical model while adjusting for study behaviors, providing a more comprehensive understanding of their relative contributions.

These findings are consistent with prior studies in medical and health professions education, showing that students who adopt more active and generative approaches tend to achieve excellent academic performance [23–25]. It is also possible that higher-performing students may be more likely to use effective learning strategies and be more aware of their use, which may contribute to reporting bias.

When individual strategies were considered, spaced practice, elaboration, and dual coding were reported more frequently by students with excellent academic performance. Each of these strategies requires active cognitive involvement and supports learning over time. Spaced practice, which involves distributing learning sessions over time rather than concentrating them in short periods, has been consistently associated with improved long-term retention in prior educational research [1,13]. Elaboration has been associated with deeper understanding by relating new information to existing knowledge and has been shown to support meaningful learning in complex educational settings [1,13,26,27]. Dual coding has been associated with improved understanding by combining verbal explanations with visual representations [1,13]. Rereading was reported frequently by students with excellent academic performance. However, passive strategies as a group were not independently associated with excellent academic performance in adjusted analyses. Thus, reliance on familiar but less efficient strategies may be less effective in supporting learning in demanding educational environments [28]. These strategies received a low utility assessment by Dunlosky et al. [10] for several reasons. Summarization has been found to benefit certain students on specific tasks. The keyword mnemonic strategy can be challenging to apply in some learning contexts and seems to offer benefits mainly for a narrow range of material and over short retention periods. Although rereading and highlighting are among the most reported study strategies, evidence suggests that they do not consistently improve academic performance, suggesting that alternative, more active learning approaches may be associated with improved learning [13]. The higher frequency of rereading among excellent performers, however, may indicate that it plays a complementary role when used alongside active strategies, rather than reflecting its effectiveness as a primary study approach.

Our findings on the association between academic performance and study behaviors are consistent with prior findings. Maintaining structured, consistent study schedules has been linked to better academic outcomes, whereas greater reliance on surface learning has been associated with poorer academic performance [10]. Our findings suggest that surface learning strategies may be beneficial when used alongside deeper approaches, rather than in isolation. These findings underscore the importance of adopting more organized study patterns and the dual cognitive demands of medical education.

High academic performers often report using organizational behaviors, such as time-management tools like planners and digital task lists, to structure daily or weekly tasks, and reported starting exam preparation earlier. However, these behaviors were not independently associated with excellent academic performance after adjustment. These findings suggest that not all commonly used study behaviors are equally effective in supporting academic performance. There were also differences in the prioritization of resources before exams across academic performance groups. Students with lower academic performance used online videos and tutorials more frequently than higher-performing students. Online resources are freely available and easily accessible, and students increasingly combine videos with other educational materials to meet their learning requirements [29,30]. However, using videos extensively immediately before exams may be less effective for exam preparation. In contrast, lecture slides and instructor-provided materials may be more closely aligned with course objectives and assessment expectations.

Examining students’ learning strategies can provide a practical basis for academic mentoring and learner support. The role of educators in guiding students to develop effective learning strategies is essential for knowledge transfer and academic success [31]. Academic monitors may use this information, particularly in the early phases of medical training, to guide students in developing appropriate learning-management skills and in selecting learning strategies that align with the educational demands of the medical curriculum.

Curriculum design plays an important role in fostering effective learning behaviors by acknowledging that successful learning in medical education requires a balance between deep and surface learning approaches rather than exclusive reliance on one. While deep learning strategies, such as elaboration and meaningful integration of knowledge, are essential for clinical reasoning and long-term retention, surface learning strategies, including memorization and factual recall, remain necessary for mastering foundational content and meeting the knowledge demands of assessments [32–34]. Teaching and assessment practices strongly influence how students combine these approaches. For example, traditional lecture-based teaching may emphasize knowledge transfer and passive learning, which is associated with reduced learning efficiency and limited development of clinical thinking and skills [35–37]. However, low-stakes quizzes administered by the educator may support student engagement, strengthen memory consolidation, and promote learning of new materials [1,38]. Curricula that incorporate spaced review of core knowledge, opportunities for elaboration through problem-solving and discussion, visual and verbal integration to support dual coding, and frequent low-stakes assessments that promote retrieval practice may support effective learning behaviors throughout medical training [33,34,38,39].

This study has several limitations. Given the cross-sectional design, these associations should not be interpreted as causal. As all variables were measured using self-reports at a single time point, common method bias may have influenced the observed associations. Reverse causality is also possible, as higher-performing students may be more likely to adopt or report effective learning strategies. Important variables such as prior academic achievement, motivation level, and socioeconomic background were not directly measured and may act as confounders influencing the observed associations. In addition, the study was conducted at a single institution, which may limit the generalizability of the findings to other educational settings with different curricula, student populations, or learning environments. The relatively low response rate of approximately 14% may also limit representativeness, as participating students may differ systematically from non-respondents, and some degree of nonresponse bias cannot be excluded. The anonymous design also prevented verification of respondent identity and duplicate submissions.

Academic performance was self-reported and could not be verified against institutional records. However, students reported their performance using the official JUST grading categories, which are clearly defined and familiar, reducing the likelihood of substantial recall errors. Any random misclassification would be expected to attenuate rather than inflate the observed associations. Retrospective self-reporting of study behaviors may also be subject to recall bias.

## Conclusion

Evidence-based learning strategies, structured study patterns, and a balance between surface and deep learning were associated with excellent academic performance among medical students. While causal relationships cannot be established, these findings highlight the potential importance of promoting active, evidence-based learning approaches within medical education. Integrating such strategies into academic mentoring and curriculum design may support more effective learning.

## Supporting information

S1 File Study questionnaire. (PDF)

S2 File Dataset. (XLSX)

## Abbreviations

JUST: Jordan University of Science and Technology
IRB: local Institutional Review Board.
