# Correction: Learning strategies, study behaviors, and academic performance among medical students in Jordan: A cross-sectional study

**DOI:** 10.1371/journal.pone.0356203

**Published:** 2026-08-14

**Authors:** Wafaa A. Mahmoud, Ahmad A. Alrawashdeh, Khaled A. Mahmoud, Nasr N. Alrabadi, Ibrahim M. Hoja, Abdelrahman M. Qasaymeh, Abdulla M. AlKofahi, Mohammad M. Alsmadi, Basel A. Alnajjar

The Funding statement for this article is incorrect. The correct Funding statement is as follows: This work was conducted under an institutional non-funded research project registered with the Deanship of Research, Jordan University of Science and Technology (Grant No. 20250401).
