# Supplementary material for: Learning strategies, study behaviors, and academic performance among medical students in Jordan: A cross-sectional study
Source: PLoS One. 2026 Jun 10;21(6):e0348623. doi: 10.1371/journal.pone.0348623 (PMC13252837; doi:10.1371/journal.pone.0348623)
Supplement: S1 File — (PDF) [file pone.0348623.s001.pdf]

English-language version of the study questionnaire

Section 1: Demographics and Background

1. **What is your current year of study?**

A) First year

B) Second year

C) Third year

D) Fourth year

E) Fifth year

F) Sixth year

2. **What is your gender?**

A) Male

B) Female

3. **What was your academic performance in the most recent academic year (for second to sixth-year students) or last semester (for first-year students)?**

A) Excellent (A+ or A)

B) Very good (B)

C) Good (C)

D) Fair (D)

E) Poor (F)

Section 2: Preferred Study Methods (Learning Strategies)

4. **Please indicate how often you use the following learning strategies:**

Learning strategy	Often	Sometimes	Rarely
Spaced practice: Implementing a schedule of practice that spreads out study activities over time.			
Interleaving: implementing a schedule of practice that mixes different kinds of problems, or a schedule of study that mixes different kinds of material, within a single study session.			
Retrieval practice: Bringing learned information to mind from long-term memory, such as self-testing or taking practice tests over to-be-learned			

material			
Elaboration: Asking and explaining why and how things work.			
Concrete examples: When studying abstract concepts, illustrating them with specific examples.			
Dual coding: Combining words with visuals.			
Summarization: Writing summaries (of various lengths) of to-be-learned texts.			
Highlighting/underlining: Marking potentially important portions of to-be-learned materials while reading.			
Keyword mnemonic technique: Using keywords and mental imagery to associate verbal materials.			
Rereading: Restudying text material again after an initial reading.			

5. **When studying, what is your primary focus?**

A) Understanding concepts deeply

B) Memorizing key facts

C) Preparing for exams specifically

D) Balancing both understanding and memorization

Section 3: Time Management Practices

6. **How many hours per day do you study during preclinical years?**

A) Less than 2 hours

B) 2–4 hours

C) 4–6 hours

D) More than 6 hours

7. **How many hours per day do you study during clinical years?**

A) Less than 2 hours

B) 2–4 hours

C) 4–6 hours

D) More than 6 hours

E) Not applicable (I am in preclinical years)

8. **Which time management technique do you use most often?**

   A) To-do lists or planners: Creating daily or weekly lists of tasks and organizing them in a planner or digital tool.

   B) Pomodoro technique: Breaking study time into intervals (e.g., 25 minutes of work followed by a 5-minute break).

   C) Prioritization of topics: Sorting tasks by importance to focus on high-priority items first.

   D) No specific strategy: Studying or completing tasks without a structured time management system

9. **How do you usually study?**

A) Regularly during the semester

B) Intensively before exams

C) Both regularly and intensively

D) Irregularly

Section 6: Exam Preparation Behaviors

10. **How early do you start preparing for major exams?**

A) More than a month in advance (continuous preparation)

B) 2–4 weeks in advance

C) 1–2 weeks in advance

D) Less than a week before

11. **What resources do you prioritize when preparing for exams?**

A) Lecture slides and notes

B) Textbooks

C) Question banks

D) Online videos or tutorials
